# Sofpironium bromide: a novel solution in the battle against primary axillary hyperhidrosis

**DOI:** 10.1097/MS9.0000000000003093

**Published:** 2025-02-28

**Authors:** Syeda Rashmeen, Iza Sohail, Maria Mohsin, Diya Anwar, Solay Farhat, Syeda Shahnoor, Adeena Maryyum, Abdul Moiz Khan

**Affiliations:** a Department of Internal Medicine, Jinnah Sindh Medical University, Karachi, Pakistan; b Department of Internal Medicine, Ayub Medical College, Abbottabad, Pakistan; c Lebanese University, Faculty of Medical Sciences, Beirut, Lebanon; d Department of Internal Medicine, Dow University of Health Sciences, Karachi, Pakistan

*Dear Editor,*

Hyperhidrosis is a chronic autonomic disorder characterized by excessive sweating exceeding thermoregulatory needs with a global prevalence of 2.8% to 18.4%^[1]^. 93% of cases are reported to be primary hyperhidrosis, of which 90% are focal, affecting the axilla, palms, soles, and craniofacial areas^[2]^. Primary axillary hyperhidrosis (PAH) attributed to sympathetic overactivity, results in excessive underarm sweating^[1]^. Contributing factors include medications, genetic predisposition, systemic diseases, hormonal fluctuations, nervousness, or stress^[2]^. PAH significantly impacts patient’s social, physical and emotional well-being, causing psychological distress^[1]^.

So far, there have been five FDA-approved options for the treatment of PAH. The details of these five FDA-approved drugs are provided in Table 1. The first line treatment option for PAH has remained topical antiperspirants (aluminum chloride solutions), which block the eccrine sweat gland duct lumen by forming precipitates^[3]^. Previously considered a last resort, anticholinergic drugs are now rising in popularity due to increased compared efficacy, particularly in topical forms. Topical anticholinergic glycopyrronium tosylate gained FDA approval in 2018 for PAH therapy while trials have been conducted on 1% and 2% glycopyrronium bromide (GPB)^[4]^. Still, treatments like iontophoresis with the DermaDry device can cause side effects such as small blisters and itching. Similarly, the MiraDry System, which uses microwave technology, may lead to underarm swelling and tenderness lasting for several days. Botulinum toxin A is an option for extremely severe PAH cases, offering a minimally invasive treatment, though the pain from injections often limits its use^[5]^. Surgical procedures, such as the removal of axillary sweat glands by curettage or liposuction, also present possible complications^[5]^. Despite the availability of diverse treatment options, each has its own limitations, underscoring the need for the exploration of new drug alternatives.

**Table 1:** Summary of FDA approved drugs for Primary Axillary Hyperhidrosis

Drug	Date of approval	Class	Mode of administration	Clinical Trial	Key Adverse Effects
OnabotulinumtoxinA	July, 2004	Botulinum toxin	Intradermally once in 12-16 weeks	2 randomized trials (PMID: 11557704 & PMID: 17306417)	Discomfort at injection site, headache
MiraDry System	February, 2011	Microwave technology.	Microwaves applied in 2 sessions with a gap of 2 weeks	Randomized, sham-controlled clinical trial (PMID: 22289389)	Redness, bruising, or numbness in the armpits
Glycopyrronium	June, 2018	Anticholinergic drug	Topically once daily on both axillae	Phase 3 trials (NCT02530281 & NCT02530294)	Blurred vision, dry mouth, drowsiness
Brella	April, 2023	Targeted alkali thermolysis (TAT) technology.	Single-use disposable sodium patch applied for approximately 3 minutes.	Phase 3 SAHARA trial (NCT04599907)	Skin abrasion and sensitivity in the armpits
DermaDry	February, 2020	Iontophoresis device	Transdermal current applied to enhance permeation of skin.	Randomized clinical trial (PMID: 31781610)	Skin irritation, muscle numbness

Recently, on 18 June 2024, 12.45% sofpironium bromide (SB), an anticholinergic drug optimized through retro-metabolic design^[6]^, received FDA approval for PAH, marking a significant milestone in advanced treatment options^[7]^. Retro-metabolic drug design incorporates metabolically sensitive moieties into drug structures, enabling rapid inactivation into non-toxic metabolites after achieving the desired therapeutic effect. This approach enhances localized efficacy, reduces systemic exposure, and minimizes adverse side effects, improving the drug’s safety and tolerability^[6]^. SB, a modified form of glycopyrronium bromide, is designed to undergo hydrolytic deactivation, thereby minimizing adverse drug reactions (ADRs). By selectively targeting the muscarinic acetylcholine receptor subtype M3, SB provides a localized therapeutic effect while reducing the ADRs commonly seen with traditional anticholinergic drugs^[6]^.

This drug was approved following the successful completion of the CARDIGAN-1 and CARDIGAN-2 clinical trials. A randomized, vehicle-controlled, multicenter CARDIGAN-1 trial was conducted with a total of 350 participants: 173 patients in the SB 15% study group and 177 patients in the vehicle group^[8]^. Similarly, the CARDIGAN-2 trial involved 351 participants, with 180 patients in the SB 15% group and 171 patients in the vehicle group^[8]^.

In both the CARDIGAN-1 and CARDIGAN-2 trials, eligible participants were male or female subjects aged 9 years or older and were required to demonstrate the ability to understand and follow all study-related procedures, including study drug administration. Included subjects had a history of PAH for 6 months or more and a total sweat production of at least 150 mg from both axillae within 5 minutes. Additionally, participants needed to have a Hyperhidrosis Disease Severity Measure-Axillary, 7-item scale (HDSM-Ax-7) score of 3 or greater. Exclusion criteria included any skin or subcutaneous tissue conditions affecting the axillae, prior use of cholinergic drugs such as bethanechol within 28 days, and known hypersensitivity to glycopyrrolate, anticholinergics, or any components of the topical formulation.

Results showed a significant improvement in the co-primary endpoints from baseline to day 43. A 2-point reduction or more in the HDSM-Ax-7 scale was observed in 49% of patients in the SB group of CARDIGAN-1 trial while 64% of patients in the SB group of CARDIGAN-2 trial saw similar results. Additionally, there were notable decreases in the median values of gravimetric sweat production (GSP), with a median reduction of 128 (201, 52) mg in the SB group of the CARDIGAN-1 trial and 143 (260, 75) mg in the SB group of the CARDIGAN-2^[8]^.

Overall, the efficacy and tolerability of SB were found to be well preserved in both the trials. SB has adverse effects typical of anticholinergic drugs, such as blurred vision, dry mouth, mydriasis, urinary hesitation, constipation, and dry eye. Additionally, local site reactions, including burning, dryness, scaling, and erythema, were more frequently observed with its use. However, most adverse events were mild or moderate and resolved after treatment cessation^[6]^. Unlike other options, it does not cause side effects such as small blisters from Iontophoresis, or swelling and tenderness from MiraDry, and it also avoids the pain associated with Botulinum toxin A injections and the complications of surgical procedures. The findings from both clinical trials are summarized in Table 2.

**Table 2:** Summary of clinical trials evaluating Sofpironium bromide efficacy

Trial	Design	Number of participants	Intervention	Control	Duration	Results
CARDIGAN-1 (NCT03836287)	Phase 3, quadruple blinded, placebo controlled, randomized clinical trial	350	Sofpironium bromide, 15% gel	Vehicle gel	6 weeks	49% of patients achieved a reduction of 2 points or more on the HDSM-Ax-7 scale and the median values of GSP decreased by 128 (201, 52) mg
CARDIGAN-2 (NCT03948646)	Phase 3, quadruple blinded, placebo controlled, randomized clinical trial	351	Sofpironium bromide, 15% gel	Vehicle gel	6 weeks	64% of patients in the SB group achieved a reduction of 2 points or more on the HDSM-Ax-7 and the median values of GSP decreased by 143 (260, 75) mg

The approval of SB marks a new era in the management of PAH. By alleviating the burden of excessive sweating, it empowers individuals to lead more fulfilling and confident lives. While SB offers an effective solution, its high cost and limited accessibility may pose challenges. Ongoing research is essential to address these barriers and further improve treatment options. Henceforth, it is of utmost importance to consider its affordability and accessibility to the general public. Owing to the high prevalence of this disease, it is crucial not only to spread awareness about the first-of-its-kind treatment option for PAH but also for government policies and planning to focus more on treating these patients first and foremost. Poor quality of life is an enormous problem for patients with PAH, and each individual should have equal access to primary care and treatment. Future research should focus on the long-term safety of SB in diverse populations, cost-effectiveness studies, and comparative trials with existing treatment options.

## Data Availability

Not applicable.
